# Prediction and characterization of human ageing-related proteins by using machine learning

**DOI:** 10.1038/s41598-018-22240-w

**Published:** 2018-03-06

**Authors:** Csaba Kerepesi, Bálint Daróczy, Ádám Sturm, Tibor Vellai, András Benczúr

**Affiliations:** 1 Institute for Computer Science and Control (MTA SZTAKI), Hungarian Academy of Sciences, Budapest, Hungary; 2 Department of Genetics, Eötvös Loránd University, Budapest, Hungary; 3 MTA-ELTE Genetics Research Group, Eötvös Loránd University, Budapest, Hungary

## Abstract

Ageing has a huge impact on human health and economy, but its molecular basis – regulation and mechanism – is still poorly understood. By today, more than three hundred genes (almost all of them function as protein-coding genes) have been related to human ageing. Although individual ageing-related genes or some small subsets of these genes have been intensively studied, their analysis as a whole has been highly limited. To fill this gap, for each human protein we extracted 21000 protein features from various databases, and using these data as an input to state-of-the-art machine learning methods, we classified human proteins as ageing-related or non-ageing-related. We found a simple classification model based on only 36 protein features, such as the “number of ageing-related interaction partners”, “response to oxidative stress”, “damaged DNA binding”, “rhythmic process” and “extracellular region”. Predicted values of the model quantify the relevance of a given protein in the regulation or mechanisms of the human ageing process. Furthermore, we identified new candidate proteins having strong computational evidence of their important role in ageing. Some of them, like Cytochrome b-245 light chain (CY24A) and Endoribonuclease ZC3H12A (ZC12A) have no previous ageing-associated annotations.

## Introduction

Genetic analysis of mortality rate has clearly revealed that ageing has strong genetic components^1–4^. Previously identified ageing-related genes are summarized in GenAge, a high quality, manually curated database^5^. The human section of GenAge (version 18) consists of 305 ageing-related genes. This set of genes includes a few members that are directly linked to human ageing, as well as the best candidate genes are supported by evidence from model organisms, using cellular experiments and functional analyses (see http://genomics.senescence.info/help.html#genage).

Features that distinguish ageing-related genes from the set of remaining human genes (hereafter referred to as “non-ageing-related” genes) may help us better understand the mechanism and regulation of the human ageing process as a whole. It was shown that ageing-related proteins, compared to non-ageing-related ones, tend to have *(i)* more protein-protein interaction (PPI) partners, *(ii)* higher K-core values (K-core is a network centrality measure defined in the Methods section), *(iii)* more ageing-related protein-protein interaction partners, and *(iv)* higher co-expression coefficients with other genes^6^.

In the present study, we analyzed not only the co-expression and protein-protein interaction features but also thousands of other protein features. Moreover, we searched not only one-variable differences between ageing-related proteins and non-ageing-related proteins but, using machine learning, we found a multi-variable model that explains what makes a protein ageing-related.

Machine learning is a rapidly growing field of computer science, in which we construct algorithms that can learn from and make predictions on data. Machine learning has many applications for science and technology^7^, including genetics and genomics^8^. Here, we applied supervised machine learning to fit a classification model of the protein features to the set of known ageing-related and non-ageing-related proteins, in order to predict ageing-related proteins and, at the same time, to understand ageing-related properties of the proteins.

A few dozen ageing studies have applied supervised machine learning methods^9^, some of them based on the GenAge database (as in the present study). Support-vector machine (SVM), k-nearest neighbour (KNN), and decision tree classifiers were used for predicting ageing-related genes of the nematode (*Caenorhabditis elegans*), fruit fly (*Drosophila melanogaster*), and mouse (*Mus musculus*) genomes^10–12^. Furthermore, a new feature selection method was constructed for the Bayesian network classifier and applied for predicting pro- or anti-longevity effects of genes of the most important model organisms^13^.

For human genes, naïve Bayes classifier and J48 decision tree were used to classify human DNA repair genes as ageing-related or non-ageing-related^14^. To our knowledge, only one study applied supervised machine learning using the whole set of human protein-coding genes^6^. Here we made several improvements on the methods of that pioneering study. For example, as databases have been extended in the last 7 years, we could use 304 ageing-related genes (from GenAge) instead of 140. We extracted not only 5 but 21000 protein features, and applied not only 280 but all of the 20183 proteins for every single training. Hence, our improved methodology has yielded new insights for ageing-related proteins.

We applied three state-of-the-art machine learning tools, XGBoost (a scalable tree boosting system^15^), logistic regression (a regression analysis of binary sequences^16^), and support-vector machine (a binary classifier for training data that are linearly non-separable^17^), to classify human proteins as ageing-related or non-ageing-related. The models are built based on 21000 protein features extracted from different databases (UniProt^18^, Gene Ontology^19^ and GeneFriends^20^), and fit to known ageing-related human proteins (extracted from GenAge^5^). The models are built from the full set of human proteins in Swiss-Prot, using the proteins included in the GenAge database as instances of the ageing-related class and all other human proteins in Swiss-Prot as the instances of the non-ageing-related class. Through this process, we uncovered the characteristic ageing-related features of human ageing-related proteins and quantified the relevance of a given protein in the regulation of the human ageing process as well as we predicted new ageing-related protein candidates.

We trained and tested our predictive methods as follows. First, we labelled every protein as an aging-related or non-aging-related protein on the basis of existing annotation in GenAge. Second, we selected a machine learning algorithm along with a fixed parameter setting. We then applied a 5-fold cross-validation, in which we split the data into 5 random parts and in each fold (round), used 4 parts to train the machine learning method and evaluated the prediction on the fifth one. Prediction for a protein is a real number between 0 and 1. At the end of the 5 fold-cross-validation, we have predicted values for the entire set of proteins, which ranks the proteins from weakest to strongest expected aging-relatedness. Then we compared the predicted values to the labels to assess prediction accuracy. Based on statistical accuracy measurements, we may compare the combination of algorithms and parameters to select the best performing method. The final prediction used to quantify the relevance of a given protein in the regulation of the human ageing process as well as to identify new ageing-related protein candidates. For more details of our method see the Methods section.

## Results

### A simple model to classify human proteins into ageing-related or non-ageing-related classes

One of our main results is a simple model with a high prediction performance that applies only 36 protein features (listed in Table 1). The model was built by using gradient boosted trees^15,21^, for feature selection and training, as described in the Methods section. This simple model shows the most important features of the classification and provides an insight into the role of the individual protein features in the regulation of the ageing process.

**Table 1:** A simple model, produced by tree boosting (XGBoost), to classify human proteins as ageing-related or non-ageing-related.

feature ID	description of the feature	category	score	relative frequency in ageing/non-ageing
ageing_n_0	number of ageing-related neighbours = 0	Net	−2.896	38.8/92.1
ageing_n_1	number of ageing-related neighbours = 1	Net	−2.275	15.8/5.6
ageing_n_2	number of ageing-related neighbours = 2	Net	−1.168	15.1/1.4
ageing_n_3_4	number of ageing-related neighbours = 3,4	Net	−0.744	12.8/0.6
GO:0043567	regulation of insulin-like growth factor receptor signaling pathway	BP	1.327	2.6/0.1
GO:0006979	response to oxidative stress	BP	0.9	21.7/1.4
GO:0003684	damaged DNA binding	MF	0.837	8.6/0.2
GO:0009987	cellular process	BP	0.805	99.3/70.0
GO:0005576	extracellular region	CC	0.636	21.7/8.8
GO:0065008	regulation of biological quality	BP	0.563	60.2/14.9
GO:0051276	chromosome organization	BP	0.515	14.5/1.6
GO:0032502	developmental process	BP	0.497	69.4/22.5
GO:0043066	negative regulation of apoptotic process	BP	0.474	32.9/3.5
GO:0009628	response to abiotic stimulus	BP	0.441	38.2/4.4
GO:0007169	transmembrane receptor protein tyrosine kinase signaling pathway	BP	0.413	19.1/2.1
GO:0010332	response to gamma radiation	BP	0.411	8.6/0.1
GO:0019838	growth factor binding	MF	0.405	5.3/0.4
GO:0040008	regulation of growth	BP	0.398	22.0/2.8
GO:0044710	single-organism metabolic process	BP	0.388	42.1/15.4
GO:0031325	positive regulation of cellular metabolic proc	BP	0.331	64.8/12.8
GO:0050896	response to stimulus	BP	0.288	77.3/22.8
GO:0031667	response to nutrient levels	BP	0.285	16.8/1.5
GO:0005515	protein binding	MF	0.271	75.7/24.4
GO:2000377	regulation of reactive oxygen species metabolic process	BP	0.259	13.8/0.6
GO:0051716	cellular response to stimulus	BP	0.257	62.2/11.1
GO:0005654	nucleoplasm	CC	0.235	49.7/14.1
GO:0080135	regulation of cellular response to stress	BP	0.225	27.3/2.6
GO:0048511	rhythmic process	BP	0.224	15.1/1.2
GO:0044427	chromosomal part	CC	0.197	24.0/3.4
ageing_n_5+	number of ageing-related neighbours ≥ 5	Net	0.192	17.4/0.2
GO:0003682	chromatin binding	MF	0.171	17.1/2.1
GO:0006974	cellular response to DNA damage stimulus	BP	0.167	27.6/3.1
GO:0097159	organic cyclic compound binding	MF	0.166	62.8/28.8
GO:0005739	mitochondrion	CC	0.16	20.4/6.1
GO:0019899	enzyme binding	MF	0.128	39.8/6.8
GO:0009894	regulation of catabolic process	BP	0.125	25.7/3.4

Features are listed by ID and description. Feature category can take values *“Net”* (Network), *“MF”* (Molecular Function), *“CC”* (Cellular Component), or *“BP”* (Biological Process). The table consists of only binary (true or false) features. For each protein we can compute the predicted relevance of ageing as follows: for each row of the table, we check whether the given feature is true for the protein and then we add up the corresponding scores. The larger the final sum, the more important role of a protein is predicted in the human ageing process. For example, suppose that a protein has 3 ageing-related neighbours and their UniProt record contains only two GO terms, “response to oxidative stress”, and “regulation of growth”. Then the predicted ageing relevance of that protein is − 0.744 + 0.9 + 0.398 = 0.554. Predicted scores produced by the above summation method are presented in the “Table1_pred” column of Supplementary Table S1. Scores obtained by summation are not necessarily bounded by 1. The actual output of XGBoost, which we used in the rest of the paper, was normalized to take values in [0…1]. In fact, we use the average of normalized predicted values made by several models (see the Methods). The relative frequency of features in the ageing-related and the non-ageing-related sets of proteins, a value independent of our particular model, is displayed in the last column.

The model (Table 1) contains only binary (true or false) features. For each human protein, we can compute the predicted relevance of ageing as follows: for each row of the table, we check whether the given feature is true for the protein, and then we add up the corresponding scores. The larger the final sum, the more important the protein is in the human ageing process by the model. Only the features that are listed in Table 1 can increase or decrease the ageing relevance score, hence these are the most important features in the human ageing-process by the model.

The results of Table 1 can be interpreted as follows. In general, the most important types of features are the features representing information about the number of ageing-related neighbours in the PPI network, which is consistent with earlier findings demonstrating that human ageing-related proteins tend to interact with other ageing-related proteins^6^. We note that degree (number of neighbours, regardless of whether or not they are ageing-related) is not among the most important features of Table 1, because in our machine learning predictions, degree had no additional prediction power when used together with the number of ageing-related neighbours.

There are twenty-one important Gene Ontology features of the biological process (BP) category (e.g. “regulation of insulin-like growth factor receptor signaling pathway” or “response to oxidative stress”), four important Gene Ontology features of the cellular component (CC) category, “extracellular region”, “chromosomal part”, “mitochondrion” and “nucleoplasm”, and six important Gene Ontology features of the molecular function category, “damaged DNA binding”, “organic cyclic compound binding”, “enzyme binding, “growth factor binding”, “protein binding” and “chromatin binding”. The fact that all of the molecular function features are binding type is consistent with the importance of the number of ageing-related neighbours.

Table 1 also shows that most of the features (32 of the 36) have a positive score, hence their existence in proteins indicates ageing-relatedness. Contrary, the existence for other features (4 of the 36 with negative scores: “ageing_n_0”, “ageing_n_1”, “ageing_n_2”, “ageing_n_3_4”) is an indicator of the non-ageing-related class.

### Human proteins with the highest predicted relevance in ageing

Sorting human proteins by predicted relevance in the regulation of the ageing process can help find the most promising targets for pharmacological or other interventions to extend human healthy lifespan. Table 2 shows the 20 most relevant ageing-related proteins we obtained by performing 20 predictions for each, by applying three different methods (XGBoost, SVM and logistic regression – see the Methods section) on the final feature set that was selected by XGBoost and sorted by the average of the predicted scores. The process is described in detail in the Methods section. Supplementary Table S1 displays a more detailed list of the predicted ageing relevance of all human proteins.

**Table 2:** Human proteins with the highest predicted relevances in ageing.

Uniprot ID	recommended name in UniProt	ageing neighbours	“aging” GO	GenAge	average predicted value
BCL2_HUMAN	Apoptosis regulator Bcl-2	4	yes	yes	0.981
FOXO1_HUMAN	Forkhead box protein O1	4	no	yes	0.96
ERCC1_HUMAN	DNA excision repair protein ERCC-1	3	yes	yes	0.944
PCNA_HUMAN	Proliferating cell nuclear antigen	4	no	yes	0.936
FOXO3_HUMAN	Forkhead box protein O3 {ECO:0000305}	5	yes	yes	0.929
SIR2_HUMAN	NAD-dependent protein deacetylase sirtuin-2	2	no	no	0.909
PTEN_HUMAN	Phosphatidylinositol 3,4,5-trisphosphate 3-phosphatase and dual-specificity protein phosphatase	5	yes	yes	0.882
APEX1_HUMAN	DNA-(apurinic or apyrimidinic site) lyase	2	yes	yes	0.857
HDAC2_HUMAN	Histone deacetylase 2	3	no	yes	0.849
MTOR_HUMAN	Serine/threonine-protein kinase mTOR	3	yes	yes	0.832
BECN1_HUMAN	Beclin-1	3	yes	no	0.827
AKT1_HUMAN	RAC-alpha serine/threonine-protein kinase	10	yes	yes	0.827
KPCD_HUMAN	Protein kinase C delta type	3	yes	yes	0.808
CDK1_HUMAN	Cyclin-dependent kinase 1	2	yes	yes	0.804
SYUA_HUMAN	Alpha-synuclein	2	yes	no	0.801
P73_HUMAN	Tumor protein p73	2	no	yes	0.8
PARP1_HUMAN	Poly [ADP-ribose] polymerase 1	6	no	yes	0.798
PRKDC_HUMAN	DNA-dependent protein kinase catalytic subunit	4	no	yes	0.791
ABL1_HUMAN	Tyrosine-protein kinase ABL1	6	no	yes	0.782
WRN_HUMAN	Werner syndrome ATP-dependent helicase	9	yes	yes	0.782

The 20 highest scored proteins considered the entire set of human proteins (regardless of whether or not the protein is included in the GenAge database), sorted by decreasing predicted relevance in ageing (average predicted value). Each row consists of an ID of the given protein (*“Uniprot ID”*), a description (*“recommended name in UniProt”*), the number of ageing-related protein neighbours of the given protein in the protein-protein interaction network (*“ageing neighbours”*), a statement about its assignment to the GO term “aging” (*“aging GO”*), a statement about its inclusion in GenAge (*“GenAge”*), and the average predicted value of 20 predictions of three machine learning methods each (XGBoost, SVM and LR) by using the final feature set selected by XGBoost (*“average predicted value”*). Average predicted values close to one indicate very strong predicted relevance for the human ageing process. Supplementary Table S1 is a more detailed list with all of the human proteins.

17 out of the 20 proteins in Table 2 have a record in the GenAge database with a detailed evidence of why it is selected in the database as an ageing-related member. For example, there are experimental evidence for the ageing-association of the homologues of human “forkhead box protein O1” (FOXO1) in worms^22^, fruit flies^23^, and mice^24^. Another example is the serine/threonine protein kinase (MTOR_HUMAN), the role of which in the ageing process was demonstrated in each of the main ageing models (*C. elegans*^25^, *Drosophila*^26^, yeast^27^ and mouse^28^), first shown by one of the authors of this paper. Finally, we note that “Werner syndrome ATP-dependent helicase” (WRN_HUMAN) is one of the strongest candidates for proteins influencing human ageing with direct evidence as mutation of WRN gene leads to Werner syndrome, which is characterized by premature ageing (progeria)^29^.

Whether or not a gene is annotated with the GO term “aging” (GO:0007568) is also displayed in Table 2; however, this term and its descendant terms are not used for modelling, we just display it as extra information. Interestingly, some proteins with a relatively high predicted score are not assigned to the GO term “aging”, showing the difference between the set of ageing-related proteins of GenAge and the set of proteins annotated with GO term “aging”.

**Figure 1:**
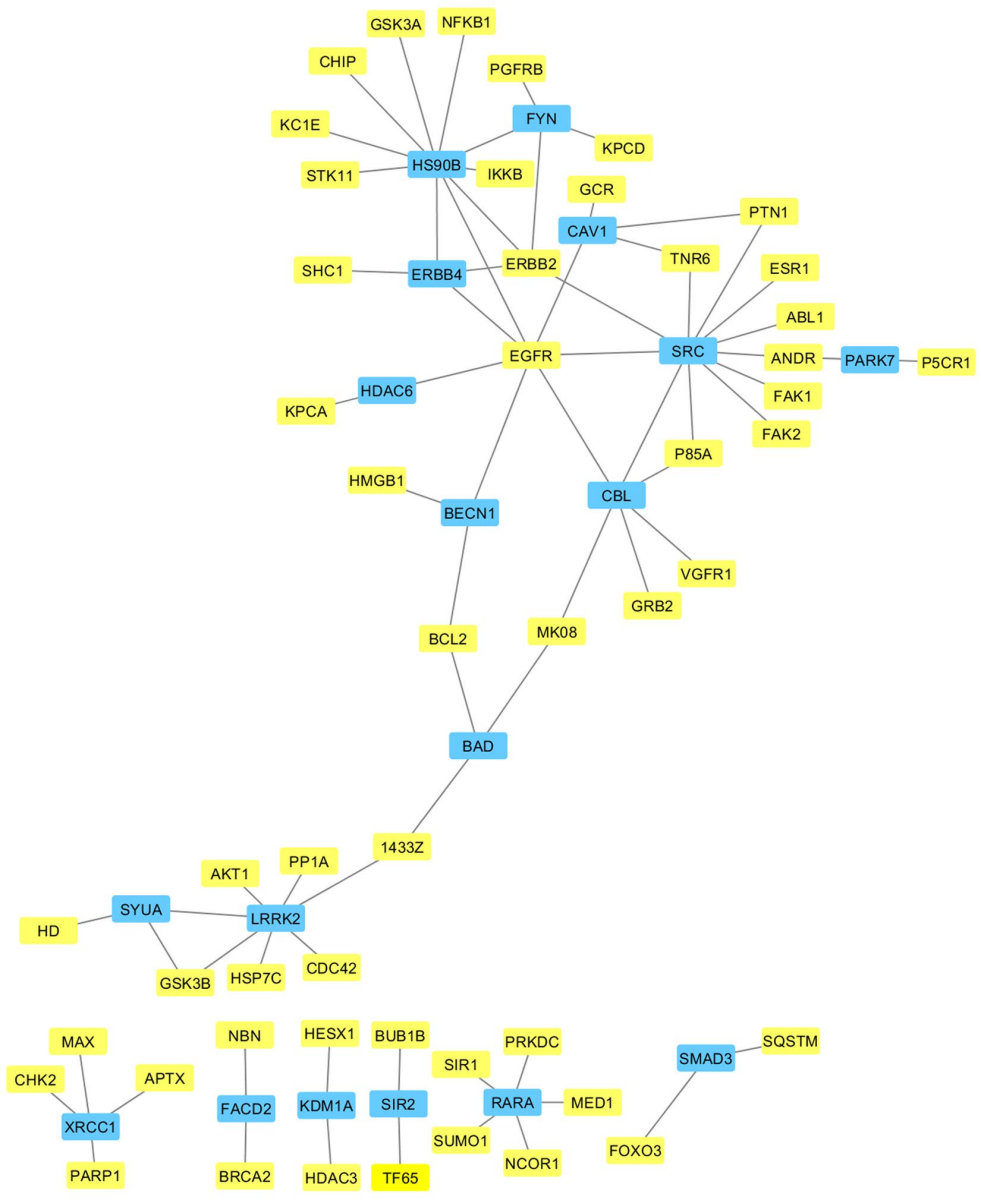
The top 20 new candidates of ageing-related proteins and their known and new ageing-related interaction partners. Blue rectangles represent the new candidates of ageing-related proteins (also listed in Table 2). Yellow rectangles represent the known ageing-related proteins of GenAge. Only the edges between yellow rectangles and blue rectangles and the edges between two blue rectangles are displayed. Nodes without edges are not displayed.

**Figure 2:**
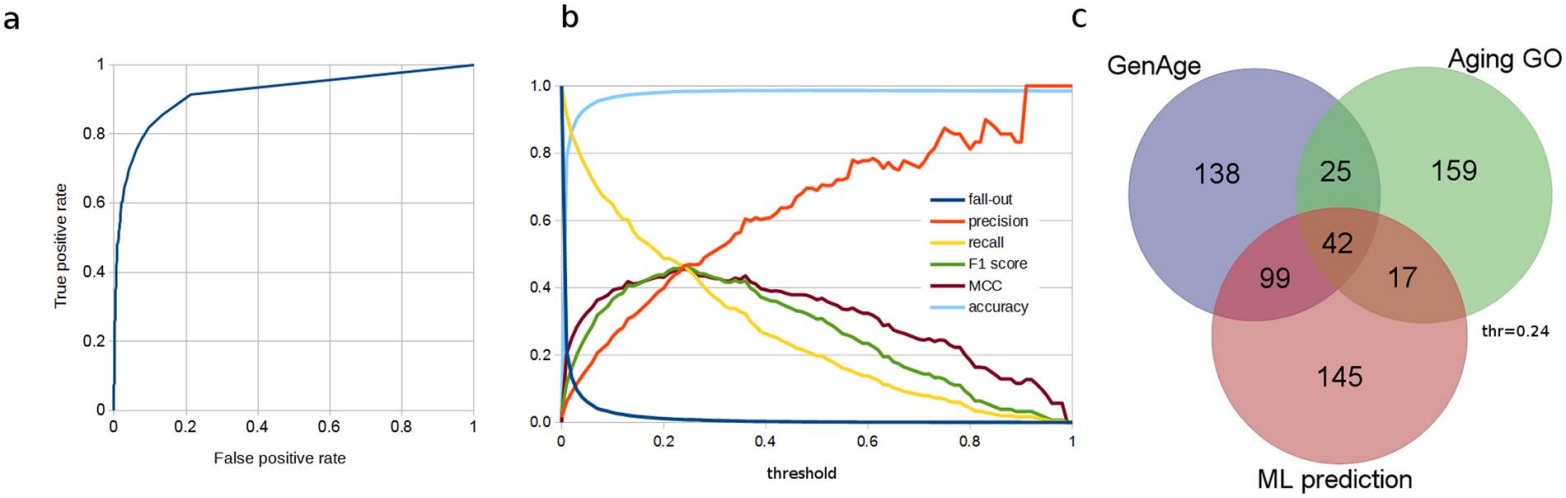
(**a**) Receiver operating characteristic curve (ROC) of our final averaged prediction (see “avg pred” in Supplementary Table S1). **(b)** Several evaluation functions calculated for different threshold values. **(c)** The number of overlapping proteins among *GenAge*, *Aging GO* (proteins annotated with the GO term “aging”) and *ML prediction* (proteins that have predicted values above the threshold 0.24).

### New candidates of ageing-related human proteins predicted by machine learning

Models we found here predict new candidates of ageing-related proteins that were previously not annotated as ageing-related in GenAge database. The 20 most promising new ageing-related candidates are listed in Table 3, and sorted by their average predicted values. The list was obtained from Supplementary Table S1 by selecting the 20 highest scored (average predicted value) proteins with no GenAge annotation. They can be considered as proteins having strong computational evidence of their regulator role in the human ageing process. Proteins highlighted in the following part of this section can be good candidates to expand GenAge database with them in the future. Table 3 contains some proteins whose counterparts have experimental evidence for regulating the ageing process in genetic model organism (BECN-1_HUMAN^30^, HS90B_HUMAN^31^).

**Table 3:** New candidates of ageing-related human proteins predicted by machine learning.

Uniprot ID	recommended name	ageing neighbours	ageing GO	GenAge	average predicted value
SIR2_HUMAN	NAD-dependent protein deacetylase sirtuin-2	2	no	no	0.909
BECN1_HUMAN	Beclin-1	3	yes	no	0.827
SYUA_HUMAN	Alpha-synuclein	2	yes	no	0.801
CAV1_HUMAN	Caveolin-1	4	no	no	0.745
LRRK2_HUMAN	Leucine-rich repeat serine/threonine-protein kinase 2	6	no	no	0.734
BAD_HUMAN	Bcl2-associated agonist of cell death	3	no	no	0.721
PARK7_HUMAN	Protein DJ-1	2	no	no	0.711
HS90B_HUMAN	Heat shock protein HSP 90-beta	8	no	no	0.709
SMAD3_HUMAN	Mothers against decapentaplegic homolog 3	2	no	no	0.662
KDM1A_HUMAN	Lysine-specific histone demethylase 1A	2	no	no	0.66
ERBB4_HUMAN	Receptor tyrosine-protein kinase erbB-4	3	no	no	0.633
HDAC6_HUMAN	Histone deacetylase 6	2	no	no	0.606
FACD2_HUMAN	Fanconi anemia group D2 protein	2	no	no	0.585
RARA_HUMAN	Retinoic acid receptor alpha	5	no	no	0.567
XRCC1_HUMAN	DNA repair protein XRCC1	4	no	no	0.567
CY24A_HUMAN	Cytochrome b-245 light chain	0	no	no	0.562
SRC_HUMAN	Proto-oncogene tyrosine-protein kinase Src	10	no	no	0.562
CBL_HUMAN	E3 ubiquitin-protein ligase CBL	5	no	no	0.561
XBP1_HUMAN	X-box-binding protein 1	0	no	no	0.551
FYN_HUMAN	Tyrosine-protein kinase Fyn	3	no	no	0.543

The 20 highest scored proteins with no ageing-related GenAge annotation, sorted by decreasing predicted relevance in ageing (average predicted value). The columns have the same meanings as in Table 2.

SIR2_HUMAN is an NAD+ (nicotinamide adenine dinucleotide)-dependent deacetylase. SIR2 overexpression has been reported to increase lifespan in *Caenorhabditis elegans* and *Drosophila melanogaster*^32,33^. Later these findings were refuted and previous, encouraging results of SIR2’s lifespan extending effect were attributed to a background mutation in the tested strains because of outcrossing of the lines with the wild type abrogated the longevity increase of SIR2 overexpression^34^. However, it was found later that the out-crossed strains are still maintained a 10–25% lifespan extension, though it was less than previously described in the original finding^35^. While the role of SIR2 in lifespan determination is still debated experimentally, our machine learning algorithms reinforced the important role of SIR2 in the ageing process (Table 3).

Caveolin-1 (CAV1_HUMAN) is a structural, scaffolding protein component of caveolae, which is an invagination of the plasma membrane enriched in cholesterol and glycosphingolipids^36^. Since it has been found that Caveolin-1 expression increases during ageing of the human prostate^37^, and the knockdown of Caveolin-1 gene accelerates the ageing process in mice^38^, it can be assumed that Caveolin-1 may have a cell protective, anti-ageing function.

LRRK2_HUMAN is a member of the leucine-rich repeat kinase family. Mutations in *LRKK2* gene are implicated in the development of Parkinson’s disease^39^. While loss-of-function mutations in *LRRK2* cause age-dependent neurodegeneration in *Drosophila*^40^, gain-of-function mutations in the gene confer resistance to age-related motor decline in mice, possibly via enhancement of LRRK2 kinase activity^41^. So, it can be assumed that LRRK2 may also have a potential neuroprotective, anti-ageing function.

Histone deacetylases (HDACs) are primarily involved in the deacetylation of histones but some HDACs, such as HDAC6_HUMAN, can also affect the function of cytoplasmic non-histone proteins. HDAC6 overexpression correlates with tumorigenesis, and improves the survival of cancer cells, which presupposes a cell protective function^42^. Indeed, the reduced expression of HDAC6 contributes to a decline in stem cell numbers^43^ and brain function^44^ during ageing. Furthermore, HDAC6 overexpression in transgenic mice increases the reproductive lifespan of animals^45^.

Additionally, we found a few proteins that have high predicted relevance in ageing but have no ageing-related annotation in GenAge, nor in the whole literature. Such proteins are Cytochrome b-245 light chain (CY24A_HUMAN) and Endoribonuclease ZC3H12A (ZC12A_HUMAN). CY24A_HUMAN is the 64th most relevant protein in ageing by our predictions (Table 3, Supplementary Table S1), and ZC12A_HUMAN is the 78th most relevant protein in ageing by our predictions (Supplementary Table S1). Neither of these proteins have ageing-related neighbours but both have 16 GO features of the 31 GO features of Table 1. The 16 ageing-related predictor features for each of these two proteins are listed in Supplementary Table S2.

Figure 1 shows how the new candidates interact with each other and with human ageing-related proteins of GeneAge. To evaluate the final prediction, we plotted the receiver operating characteristic curve (ROC, Fig. 2a). The performance of the model was 0.9322, a result we obtained by measuring the area under the curve of the receiver operating characteristic curve (ROC AUC). It is shown that ROC AUC (shortly: AUC) is the probability that a randomly chosen positive example is predicted with a higher score than a randomly chosen negative example^46^, hence AUC is independent of the class imbalance.

To compare our prediction to the ageing-related proteins of GenAge, and the set of proteins annotated with the GO term “aging”, we chose a threshold (0.24) for the predicted relevance in ageing (“avg pred” in Supplementary Table S1) (Fig. 2c); a protein is predicted as ageing-related by the models if its predicted relevance in ageing is at least 0.24. We selected this threshold because at this point, there is a relatively high *true positive rate* (0.4638) and, at the same time, a relatively low *false positive rate* (0.0081) and maximal *F1 score* (0.46458) and maximal *MCC* (0.45641) are reached at this point (*FP* = 162, *TP* = 141, *FN* = 163, *TN* = 19717, *precision* = 0.46535, *recall* = 0.46382, *accuracy* = 0.98390). Evaluation measures for more threshold values are available in Supplementary Table S3, and displayed in Fig. 2b. For definitions of the evaluation measures see the Methods section.

## Discussion

In this study, we ordered the human proteins on the basis how (to which extent) machine learning algorithms, which automatically build a classifier by learning from a set of labelled data, predict their importance in the regulation or mechanism of the ageing process. The results we obtained have at least two important relevancies. First, they may help identify the ageing-related proteins that have a particularly prominent role in the human ageing process (quantifying the importance of ageing-related proteins in the process). Second, the results may help uncover novel proteins with an ageing function (the role of these proteins in ageing has not been recognized previously). Furthermore, we created a simple, biologically easily interpretable model, based on only 36 protein features that may help to understand better the human ageing process.

Ageing is driven by the progressive accumulation of unrepaired cellular damage^4,47^. Such damages mainly include oxidized, aggregated and misfolded proteins that are generated by mutations, environmental factors (e.g. heat stress) and metabolic agents (e.g. reactive oxygen species produced by mitochondrial respiration), and act as cellular toxins often causing the loss of the affected cells^48^. At advanced ages, massive levels of cell death can lead to the development of an age-associated degenerative disease (tissue dysfunction), and eventually organismal death. Prior to this life period, cellular damages are effectively degraded (i.e. eliminated) by the repair and maintenance processes and mechanisms including autophagy (cellular self-eating) being the most significant form of breaking down cytoplasmic materials^49,50^, the ubiquitin-proteasome system and molecular chaperons, also called heat-shock proteins, as well as the DNA repair pathways. These processes and mechanisms, however, display a gradual decline in their capacity as the organism ages. In the present study, BCL2 (antiapoptotic B cell lymphoma protein), FOXO1 (Fork head box O transcription factor) and ERCC1 (DNA excision repair protein) were identified as proteins with the highest predicted relevance in human ageing (Table 2). Indeed, BCL2 protects cells from undergoing apoptosis (programmed cell death), and, in both nematodes and human cells, also interacts with the autophagic process through binding the core autophagy protein BECN1 (Beclin – Bcl2-interacting)^51^. FOXO1 operates as a downstream component of the insulin/IGF-1 (insulin-like growth factor) signalling pathway, which plays a pivotal role in the control of ageing in divergent eukaryotic organisms. ERCC1 primarily functions in DNA repair to lower the level of mutations causing cellular damage. Among the new candidate human ageing-related proteins we identified here, SIR2 (sirtuin, a NAD-dependent histone deacetylase) and BECN1 were ranked to the top of the list (Table 3). There are several lines of evidence that both proteins are implicated in the ageing process. For example, BEC-1 (Beclin homologue), the C. elegans orthologue of human BECN1, was directly implicated in lifespan determination^30^. Together, we conclude that novel ageing-related protein candidates we identified by machine learning represent mostly true hits, which can be validated by further experimental analysis.

Supervised machine learning methods are especially effective when they are used on a large set of examples. Earlier machine learning studies on human proteins applied only a few hundred features of a few hundred proteins for each training^6,14^. By using extensive computational power, here we analyzed all the human proteins, and performed feature selection from 21000 protein features. In 2016, a novel machine learning system was developed, XGBoost^15^, which allows an effective feature selection even in case of a huge number of correlating features. XGBoost is applied widely by data scientists for example at data mining challenges^15^. However, according to our knowledge, we are the first who apply it for ageing research. Boosted trees may be widely used in further analysis of this field.

We used the GenAge database^5^ to assign the human proteins into “ageing-related” or “non-ageing-related” classes in the following way: the 304 proteins of GenAge served as “ageing-related” instances and the remaining 19879 human proteins served as “non-ageing-related” instances. These classes then served as labels for training the classifiers. We applied GenAge because it focuses on the ageing process when selecting genes (see http://genomics.senescence.info/help.html#genage). Genes, however, that modulate (primarily limit) lifespan independently of the ageing process are omitted from this database. Such genes are involved in human pathologies or their activity is altered in case of extreme longevity. In addition, several other related supervised machine learning studies also rely on GenAge^6,10–14^.

One may ask why the “aging” GO annotation was not used in the process of labelling the proteins for training the classifiers. We used only GenAge for labeling for several reasons. First, GenAge has a more detailed explanation and references than the “aging” GO annotations. Second, we could find no study related to machine learning based on “aging” GO terms. Third, it seems that the “aging” GO assignment process does not focus on the regulation of the ageing process. For example, “aging” GO assignments of the proteins KRA43, KRA45, KRA47, KRA48, KRA49, K1C14, K1C16, KRT83 and KT33B are based on the single evidence that keratin and keratin-associated proteins in white hair are upregulated in comparison with black hair in microarray experiments^52^. However, using both GenAge and “aging” GO annotations would give a wider perspective of ageing. So, we performed a supplementary analysis based on a labelling where a given protein was assigned to the ageing-related class if it is included in GenAge or annotated with the “aging GO” term or its descendants. The results, methods and discussion sections of the supplementary analysis can be found in Supplementary Information, Supplementary Tables S4–S6.

It is important to emphasize that the vast majority of human ageing-related proteins, including those listed in GenAge, have not been validated experimentally for a regulator role in human ageing. Relevant results have been obtained mostly from genetic model systems and assumed that they operate in an evolutionarily conserved way. As an example, defects in the transmembrane receptor for insulin/IGF-1 signalling have been shown to double lifespan in nematodes (C. elegans)^53^ but there is no evidence for a gene/protein that can extend human lifespan in such an extreme manner. Some degree of ageing regulator evidence exists only for a few human proteins. WRN, for example, which encodes a RecQ helicase involved in DNA repair, when is mutated, leads to Werner syndrome, the pleiotropic phenotype of which is characterized by extreme progeria^29^. Prominent or novel ageing proteins we identified in this work may become promising drug targets for further efforts in order to extend healthy lifespan in humans, which is a central focus in current pharmacological research.

Despite its medical and social significance, our present knowledge on the biological basis of the (human) ageing process is rather limited. As Cynthia Kenyon wrote in one of her review articles on ageing^3^, genetic factors that primarily cause ageing (i.e. the progressive, lifelong accumulation of cellular damage) remain unexplored. Recent theoretical considerations have tried to identify a novel class and high copy number of genes, mobile genetic elements, as primary genetic determinants of ageing^54,55^, but a relevant direct experimental evidence is still missing to support this assumption. In the light of these facts and as databases are being improved considerably, our present ageing-related ordering (Supplementary Table S1) may be modified in the future.

Here we ignored an ageing-related gene, telomerase reverse transcriptase (TERT), because it does not code for a protein. An interesting future direction would be to predict not only ageing-related proteins but ageing-related non-coding RNAs. Such a work could be based on results of the computational prediction and characterization of disease-associated human microRNAs^56–59^, and long non-coding RNAs^60,61^.

## Conclusion

Although single ageing-related proteins have been intensively studied, their analysis as a whole has been largely limited. To fill this gap, in the present work, we applied three state-of-the-art machine learning tools to classify human proteins as ageing-related or non-ageing-related. The classification models are built on all human proteins and 21000 protein features, and fit to known ageing-related human proteins of the GenAge database. The models were built from the full set of human proteins in Swiss-Prot, using the proteins included in the GenAge database as instances of the ageing-related class and all other human proteins in Swiss-Prot as the instances of the non-ageing-related class. The final prediction was used to quantify the relevance of a given protein in the regulation of the human ageing process as well as to identify new ageing-related protein candidates.

## Methods

**Figure 3:**
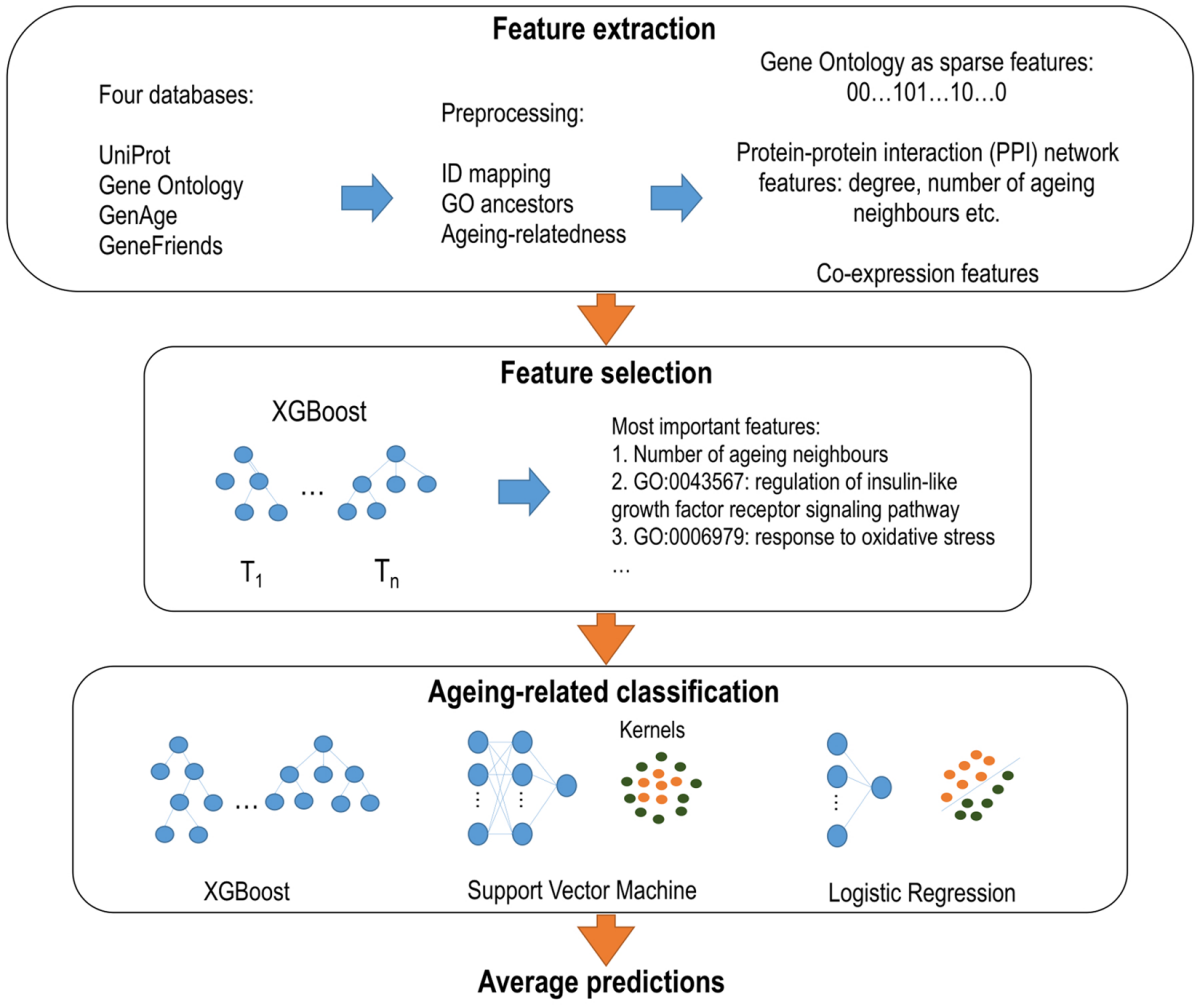
Overview of the study methods as the main ingredients of our classification method. We utilized four databases (UniProt, Gene Ontology, GenAge, GeneFriends) and after ID mapping and GO ancestor determination, we extracted several feature sets. Then we selected the most important features in several steps, which considerably reduced the dimensionality of the final feature space. Finally, we used three different classification methods (XGBoost, support vector machine, logistic regression) trained on the selected features and then we averaged the predicted values of the three methods.

We start this section by describing the source of known ageing-related proteins. We continue by describing the Gene Ontology features, the protein-protein interaction (PPI) network features and the co-expression feature. Then we detail how gradient boosted trees were applied for selecting the most relevant features. The main steps are shown in Fig. 3. We close this section by describing the best performing machine learning methods.

### Ageing-related data (labels of the classification)

All the 20183 human Swiss-Prot (manually annotated and reviewed UniProt) entries were downloaded from the UniProtKB protein database^62^ on April 6, 2017. In the human section of GenAge database (Build 18), we found 305 candidates of human ageing-related genes^5^. With the exception of a single gene TERT, all of these genes are included in Swiss-Prot. Hence, the target variable (labels) of the classification has value “1” for the 304 proteins of GenAge (“ageing-related class”) and value “0” for the remaining 19879 human proteins (“non-ageing-related” class).

### Gene Ontology features

We compute Gene Ontology (GO) features in a similar way as Freitas *et al*.^14^, but by also using the GO categories “cellular component” and “molecular function”. For each human Swiss-Prot protein entry we extract the associated GO terms, all of which are binary, being either yes or no. The difficulty of this task is that the GO assignments of the UniProt entries are not complete: an entry is associated with a given GO term but not all the ancestors containing the given GO term. For example, the ANKE1_HUMAN protein entry has only the GO term “calcium ion binding” (GO:0005509) but does not have the ancestor GO terms “metal ion binding” (GO:0046872), “cation binding” (GO:0043169), “ion binding” (GO:0043167), “binding” (GO:0043167), and “molecular function” (GO:0003674). To handle this problem, we downloaded the basic version of the Gene Ontology database (with the database filename “go-basic.obo”) and by walking upward in the GO hierarchy, we added all of the ancestor GO terms to the corresponding proteins. Note that “go-basic.obo” is guaranteed to be acyclic, and annotations can be propagated up the (directed) graph. The final feature table contains 20183 proteins and 21019 features. Although the notion of ageing-relatedness of the GenAge database is far from being identical to that of the Gene Ontology database (see Fig. 2), we removed the GO terms, along with their descendants, that contain “aging”, “senescence” or “age-related” as substring (these terms are also used by Chautard *et al*.^63^).

### PPI network features

Protein-protein interactions (PPIs) are included in the Swiss-Prot database. In our PPI network of 20183 nodes and 18784 edges, we only kept bidirectional and non-self interactions.

For each protein, we computed the following features based on the constructed network and the ageing-related data. In terms of interaction count statistics, we computed the number of neighbours, the number of ageing-related neighbours, and the ratio of the two. We also computed the K-core value^64^ of a node by using the “coreness” function of the R package igraph^65^. A K-core of a graph is a maximal subgraph in which each vertex has degree at least K. The K-core or coreness value of a node is the maximal value of K such that the node is in a K-core. We extracted further network features by Cytoscape, including “Average Shortest Path Length”, “Betweenness Centrality”, “Closeness Centrality”, “Clustering Coefficient”, “Eccentricity”, “Neighborhood Connectivity”, “Radiality”, “Stress” and “Topological Coefficient”^66,67^.

### Co-expression feature

For each human protein-coding gene, we computed its gene co-expression with the set of ageing-related genes using the GeneFriends database^20^. Co-expression is the number of human ageing-related genes of GenAge that increase or decrease in expression simultaneously in the RNAseq datasets processed by GeneFriends.

### Feature selection with XGBoost

Gradient boosted tree algorithms^21^ are capable of selecting the most important uncorrelated features by building small decision trees of a few of the most important features and gradually refining the small models by adding new trees. We used the XGBoost implementation^15^ for feature selection with the parameters shown in Table 4. We evaluated the generated models by 5-fold cross-validation and measured the area under the curve of the receiver operating characteristic curve (ROC AUC). For every feature set, we repeated this process 20 times. The average and standard deviation of the 20 predictions are shown in Table 4. In the first steps of the feature selection process we selected the most important Gene Ontology features except the GO terms related to ageing. Original Gene Ontology (GO) terms with the ageing-related terms produced an AUC of 0.8787 and 16820 features. Original Gene Ontology (GO) terms without the ageing-related terms produced an AUC of 0.8729 and 16800 features. The explanation for this surprisingly low increasing is the large difference between the set of ageing-related proteins of GenAge and the set of proteins annotated with GO term “aging” (as Fig. 2c showed). GO ancestor calculation has a considerable added value, reaching an AUC of 0.9086 and 21000 features.

**Table 4:** Feature selection process driven by performance of XGBoost on different feature sets.

short description of the feature set	number of features	depth of trees	number of trees	number of predictions	AUC	
					average	std dev
GO w/o ancestors, with ageing GOs	16820	6	20	20	0.8787	0.0061
GO w/o ancestors	16800	6	20	20	0.8729	0.0050
GO	21000	6	20	20	0.9086	0.0049
GO XGBoost one pass filter	373	6	20	20	0.9187	0.0042
GO XGBoost two pass filter	65	6	20	20	0.9219	0.0033
GO XGBoost two pass filter UniNet, CoExp	79	6	20	20	0.9294	0.0034
GO XGBoost two pass filter, UniNet	78	6	20	20	0.9293	0.0036
GO XGBoost two pass filter, degree	66	6	20	20	0.9283	0.0027
GO XGBoost two pass filter, ageing_n	66	6	20	20	0.9314	0.0029
GO XGBoost three pass filter, ageing_n	32	1	50	20	0.9322	0.0011

Performance of different feature sets, from weakest down to strongest, by comparing classification performance of 20 prediction each. Default settings for Gene Ontology (GO) features are “without ageing GOs but with GO ancestors”; we marked when used otherwise. For each feature set description (row), we list the number of features, the depth and number of trees in the model and the average and standard deviation of AUC values generated by 20 predictions of 5-fold cross-validation. *“UniNet”* means the set of network features (including degree, ageing_n, and the remaining network features), *“CoExp”* means the co-expression feature.

We used feature selection started from this set of 21000 GO features in two passes. First, we used XGBoost for selecting the GO features by computing the importance of features and selecting those with value greater than 0. We reached an AUC of 0.9187 (improvement by 0.0101) with only 373 GO features left from the initial 21000. By the second filter, XGBoost selected the GO features that have feature importance values greater than 0.004. We reached an AUC of 0.9219 with only 65 GO features left from the initial 373.

Given the 65 GO features selected in two passes by XGBoost, we continued feature selection by adding network and co-expression features. All these features produced an AUC of 0.9294, showing a considerable increase. However, we found that the filtered GO features with the addition of a single feature, the number of ageing-related neighbours (“ageing_n”) produced a slight increase in AUC (0.9314). Since simpler models usually generalize better, we kept 66 features with the 65 GO features and the number of ageing-related neighbours.

In the last step of feature selection we applied a third filter, where XGBoost (with 50 trees and maximal depth 1) selected features with importance greater than 0. At this point, we reduced the XGBoost parameter depth of tree to achieve a simple, well interpretable model (at the same time we needed to increase the number of trees to reach the same performance).

Only 32 features left from the initial 66, and we reached a final AUC of 0.9322. This final feature set was used for the predictions in the results section and it is shared at https://github.com/kerepesi/aging_ml along with codes to reproduce the results.

### Predictions via SVM and LR on the feature set selected by XGBoost

Besides XGBoost, we performed 20 predictions of 5 fold cross-validations (5 fold CV is repeated 20 times) with support vector machine (SVM)^17^ and logistic regression (LR)^16^ on the final 32 features selected by XGBoost. Logistic regression with the default parameter settings (scikit-learn, version 0.19.0^68^), produced an average AUC of 0.9279 (std dev 0.0009). SVM with linear kernel function and balanced class weight on *L*^2^ normalized feature space produced an average AUC of 0.9321 (std dev 0.0015). Average predicted values of each method are presented in Supplementary Table S1.

### Performance of various machine learning algorithms

We compared performance of XGBoost (learning rate = 0.3, depth of trees = 6, number of trees = 20) with various machine learning algorithms (with the default settings of scikit-learn, version 0.19.0^68^): k-nearest neighbour, decision tree, naïve Bayes, logistic regression, and support-vector machine with linear kernel function. Most of them appeared in related studies. We applied the algorithms on the whole set of features without selection (GO, UniNet, CoExp), as well as, on a feature set containing only the GO features that occur in at least 100 proteins (idea of occurrence threshold is inspired by Freitas *et al*.^14^). For each algorithm and feature set the average and standard deviation of AUC values generated by predictions of 5-fold cross-validation are presented in Table 5. XGBoost outperformed the remaining methods.

**Table 5:** Performance of various machine learning algorithms on two different feature sets.

short description of the feature set	name of algorithm	number of features	number of predictions	AUC	
				average	std dev
GO, UniNet, CoExp	k-nearest neighbour	21014	20	0.5614	0.0053
GO, UniNet, CoExp	decision tree	21014	20	0.6373	0.0113
GO, UniNet, CoExp	naïve Bayes	21014	20	0.7258	0.0056
GO, UniNet, CoExp	logistic regression	21014	20	0.7374	0.0538
GO, UniNet, CoExp	support-vector machine	21014	20	0.9091	0.0022
GO, UniNet, CoExp	XGBoost	21014	20	0.9201	0.0024
Frequent GOs, UniNet, CoExp	k-nearest neighbour	310	20	0.5857	0.0082
Frequent GOs, UniNet, CoExp	decision tree	310	20	0.6191	0.0095
Frequent GOs, UniNet, CoExp	naïve Bayes	310	20	0.7991	0.0025
Frequent GOs, UniNet, CoExp	logistic regression	310	20	0.8036	0.0343
Frequent GOs, UniNet, CoExp	support-vector machine	310	20	0.8739	0.0109
Frequent GOs, UniNet, CoExp	XGBoost	310	20	0.9088	0.0041

Performance of various machine learning algorithms on two different feature sets. “GO, UniNet, CoExp” means the feature set containing all GO features without ageing GOs but with GO ancestors, the network features and the co-expression feature. “Frequent GOs, UniNet, CoExp” means the feature set containing only GO features that occur in at least 100 proteins (selected from the above mentioned feature set). For each raw, we list the feature set description, the name of the algorithm, the number of features, the number of predictions, and the average and standard deviation of 20 AUC values generated by a number of predictions of 5-fold cross-validation.

### Evaluation measures for binary classification

TP (true positive) is the number of positives that are predicted as positives. TN (true negative) is the number of negatives that are predicted as negatives. FP (false positive) is the number of negatives that are predicted as positives. FN (false negative) is the number of positives that are predicted as negatives. In our context “positive” means “ageing-related”, “negative” means “non-aging-related”. *Precision*, *recall* (or *true positive rate*), *fall-out* (or *false positive rate*), *accuracy*, *F1 score* and *MCC (Matthew Correlation Coefficient)* were computed as followings:

$$precision\,:=\{\begin{array}{cc}\frac{TP}{TP+FP}, & {\rm{i}}{\rm{f}}\,TP+FP\ne 0,\\ 1, & {\rm{o}}{\rm{t}}{\rm{h}}{\rm{e}}{\rm{r}}{\rm{w}}{\rm{i}}{\rm{s}}{\rm{e}}.\end{array}\,\,\,\,\,recall\,:=\frac{TP}{TP+FN},\,\,\,\,fall{\textstyle \text{-}}out\,:=\frac{FP}{TN+FP}$$

$$accuracy\,:=\frac{TP+TN}{TP+TN+FP+FN},\,\,\,\,{F}{1}\,{s}{c}{o}{r}{e}\,:=\{\begin{array}{cc}\frac{2\cdot precision\cdot recall}{precision+recall}, & {\rm{i}}{\rm{f}}\,precision+recall\ne 0,\\ 0, & {\rm{o}}{\rm{t}}{\rm{h}}{\rm{e}}{\rm{r}}{\rm{w}}{\rm{i}}{\rm{s}}{\rm{e}}.\end{array}$$

$$MCC\,:=\{\begin{array}{cc}\frac{TP\cdot TN-FP\cdot FN}{\sqrt{(TP+FP)(TP+FN)(TN+FP)(TN+FN)}}, & {\rm{i}}{\rm{f}}\,(TP+FP)(TN+FN)\ne 0,\\ 0, & {\rm{o}}{\rm{t}}{\rm{h}}{\rm{e}}{\rm{r}}{\rm{w}}{\rm{i}}{\rm{s}}{\rm{e}}.\end{array}$$

We note that—in a binary classification task—there are at least one positive sample (i.e. *TP* + *FN* ≥ 1) and at least one negative sample (i.e. *TN* + *FP* ≥ 1), hence the denominator of the formula of *recall*, *fall-out* and *accuracy* can never be equal to zero.

*ROC curve (Receiver Operating Characteristic Curve)* is defined by the point pairs of true positive rates and false positive rates at different threshold settings. *ROC AUC* (shortly *AUC*) is calculated as the area under the *ROC curve*.

### Data and code availability

Tables and codes of the final results are available at https://github.com/kerepesi/aging_ml. Other intermediate data and codes of this study are available from the corresponding author upon reasonable request.

## Electronic supplementary material

Supplementary Information

Supplementary Table S1

Supplementary Table S2

Supplementary Table S3

Supplementary Table S4

Supplementary Table S5

Supplementary Table S6
