# Supplementary material for: Prediction and characterization of human ageing-related proteins by using machine learning
Source: Sci Rep. 2018 Mar 6;8:4094. doi: 10.1038/s41598-018-22240-w (PMC5840292; doi:10.1038/s41598-018-22240-w)
Supplement: Supplementary file 1 — Supplementary Information [file 41598_2018_22240_MOESM1_ESM.pdf]

Prediction and characterization of human ageing-related proteins by using machine learning

Csaba Kerepesi, Bálint Daróczy, Ádám Sturm, Tibor Vellai, and András Benczúr

SUPPLEMENTARY INFORMATION

Supplementary Table Legends

__Supplementary Table S1.__ **The entire set of human proteins, sorted by a decreasing predicted relevance in ageing**. The models were built using the proteins included in the GenAge database as instances of the ageing-related class and all other human proteins in SwissProt as the instances of the non-ageing-related class. The final prediction was served as a refinement of the labelling, and used to quantify the relevance of a given protein in the regulation of the human ageing process as well as to identify new ageing-related protein candidates. Each row consists of an ID of the given protein (*"Uniprot ID"*), a description (*"recommended name in UniProt"*), the number of protein neighbours of the given protein in the protein-protein interaction network (*"degree"*), the number of ageing-related protein neighbours of the given protein in the protein-protein interaction network (*"ageing neighbours"*), value "1" if the protein is associated with the GO term "aging" and value "0" otherwise (*"aging GO"*), value "1" if the protein included in GenAge and value "0" otherwise (*"GenAge"*), the predicted value calculated from the scores of the "simple model" presented in Table 1 (*"Table1_pred"*), the average predicted value of 20 predictions of XGBoost on the final feature set selected by XGBoost (*"XGBoost pred (avg of 20 preds)"*), the average predicted value of 20 predictions of SVM on the final feature set selected by XGBoost (*"SVM pred (avg of 20 preds)"*), the average predicted value of 20 predictions of LR on the final feature set selected by XGBoost (*"LR pred (avg of 20 preds)"*), and the average predicted value of 20-20 predictions of three machine learning methods, XGBoost, SVM and LR (*"avg pred (avg of the cols H, I, J)"*). Tables 2 and 3 were generated from this table.

__Supplementary Table S2.__ **GO terms associated with Cytochrome b-245 light chain (CY24A_HUMAN) and Endoribonuclease ZC3H12A (ZC12A_HUMAN) in the final model.** Final predicted values of a given protein were calculated by a summation of its *"score"* values.

__Supplementary Table S3.__ **Evaluation indicators of the final model for different threshold values** *("thr"):* fall-out or false positive rate (*"fall-out"),* precision (*"precision"*), recall or true positive rate (*"recall"*), average of precision and recall (*"(precision+recall)/2"),* precision multiplied by recall (*"precision*recall"*), F1 score or F-measure (*"F1 score"*), Matthews correlation coefficient (*"MCC"*), number of false positive instances (*"FP"*), number of true positive instances (*"TP"*), number of false negative instances (*"FN"*), number of true negative instances (*"TN"*), accuracy (*"accuracy"*).

__Supplementary Table S4.__ **As a part of the supplementary analysis, the entire set of human proteins listed by a decreasing predicted relevance in ageing:** instances of the ageing-related class consist of the proteins included in the GenAge database and even the proteins associated with the Gene Ontology term "aging" or its descendant terms. All other human proteins in SwissProt belonged to the non-ageing-related class. Each row consists of an ID of the given protein (*"Uniprot ID"*), a description (*"recommended name in UniProt"*), the number of protein neighbours of the given protein in the protein-protein interaction network (*"degree"*), the number of ageing-related protein neighbours of the given protein in the protein-protein interaction network (*"ageing neighbours"*), value "1" if the protein is associated with the GO term "aging" and value "0" otherwise (*"aging GO"*), value "1" if the protein is included in GenAge and value "0" otherwise (*"GenAge"*), the average predicted value of 20 predictions of XGBoost on the final feature set selected by XGBoost (*"XGBoost pred (avg of 20 preds)"*).

<u>Supplementary Table S5.</u> Performance of different feature sets of the supplementary analysis, from weakest down to strongest, by comparing classification performance of 20 predictions each: instances of the ageing-related class consists of proteins included in the GenAge database and also proteins associated with the Gene Ontology term “aging” or its descendant terms. We compared classification performance of 20-20 predictions with different sets of features. Default settings for Gene Ontology (GO) features are “without ageing GOs but with GO ancestors”; we marked when used otherwise. For each feature set description, we list the number of features, the depth and number of trees in the model, and the average and standard deviation of 20 AUC values generated by 20 predictions with 5-fold cross-validation.

<u>Supplementary Table S6.</u> List of the final features of the supplementary analysis: features are listed by ID and description. Feature category can take values *“Net”* (Network), *“MF”* (Molecular Function), *“CC”* (Cellular Component), or *“BP”* (Biological Process).

Supplementary analysis: machine learning prediction of ageing-related proteins based on an extended ageing-related class

In the main part of the paper, we used GenAge database to label the human proteins to “ageing-related” or “non-ageing-related” classes in the following way: 304 human proteins of GenAge served as “ageing-related” instances and the remaining 19879 human proteins served as “non-ageing-related" instances. This classification served as the basis for the main part of the paper.

Here, we extended the above mentioned 304 ageing-related proteins with those proteins which have a GO annotation related to ageing (“aging” GO or its descendants). In this way, we obtained 501 ageing-related proteins and the remaining human proteins served as “non-ageing-related" instances. Then we performed the same machine learning predictions (with some simplifications) as described in the main text but here we used the new labelling.

Result of the main predictions can be considered as an analysis of the fundamental ageing process, while supplementary analysis can be considered as an analysis of a wider perpective of ageing which included every protein (less or more) associated with ageing.

Results

Final prediction of supplementary analysis are presented in Supplementary Table S4 containg the entire set of human proteins sorted by decreasing predicted relevance in ageing (average predicted value) where the models are built on the extended ageing-related labelling: instances of the ageing-related class consists of the proteins included in the GenAge database and also the proteins associated with the Gene Ontology term “aging” or its descendant terms. All other human proteins in SwissProt were assigned to the non-ageing-related class.

The performance of the final model prediction produced an AUC of 0.9307, a result we obtained by measuring the area under the curve of the receiver operating characteristic curve (ROC AUC) and averaged the AUC values of 20 predictions of the XGBoost machine learning method on the selected final feature set (see the Methods section, below).

The most relevant ageing-related proteins according to the supplementary predictions: SIR1_HUMAN, AKT1_HUMAN, P53_HUMAN, PCNA_HUMAN and PML_HUMAN. The most relevant ageing-related proteins by the supplementary predictions with no ageing-related GenAge annotation and no aging “GO” (or its descendants) annotation: B2CL1_HUMAN, KDM1A_HUMAN, MEN1_HUMAN, PLAK_HUMAN and CCND1_HUMAN. They can be considered as proteins having computational evidence of some (more or less) association with ageing.

Methods

Ageing-related data (labels of the classification). We started from the 20183 human Swiss-Prot proteins obtained in the main predictions (see the Methods section of the main text). The target variable (labels) of the classification has the value “1” for the 501 proteins, which are included in GenAge (“ageing-related class”) or

have a GO annotation related to ageing (“aging” GO or its descendants) and the value ”0” for the remaining 19682 human proteins (“non-ageing-related” class).

PPI network features. Values of “degree” were adopted from the main results calculated (see Methods section in the main text), “ageing-neighbour” were calculated according to the actual labels.

Feature selection and predictions with XGBoost. We performed feature selection in a similar way (with some simplifications) as described in the Methods section of the main text. We evaluated the generated models of each predictions by 5-fold cross-validation and measured the area under the curve of the receiver operating characteristic curve (ROC AUC), and for every feature set we repeat this process.

We started from the Gene Ontology features without the “aging” GO and its descendants. This feature set contained 21000 features and produced an AUC of 0.9263. Then we used feature selection for this set of 21000 GO features in three passes. First, we used XGBoost for selecting GO features by computing the importance of features and selecting those with a value greater than 0. We reached an AUC of 0.9334 with only 391 GO features left from the initial 21000. By the second filter, XGBoost selected GO features that have feature importance values greater than 0.004. We reached an AUC of 0.9341 with only 70 GO features left from the initial 391. The filtered GO features with the addition of a single feature, the number of ageing-related neighbours (“ageing_n”) produced a slight increase in AUC (0.938). In the last step of feature selection, we applied a third filter, where XGBoost (with 50 trees and maximal depth 1) selected features with importance greater than 0. Only 36 features left from the initial 71, and we reached a final AUC of 0.9307. This final feature table was used for the predictions of the supplementary analysis (Supplementary Table S4), and is shared at the https://github.com/kerepesi/aging_ml/tree/master/supplementary_predictions with codes to reproduce the results. Descriptions of features of the final feature table are presented in Supplementary Table S6.
